# The Regulatory Protein ChuP Connects Heme and Siderophore-Mediated Iron Acquisition Systems Required for *Chromobacterium violaceum* Virulence

**DOI:** 10.3389/fcimb.2022.873536

**Published:** 2022-05-11

**Authors:** Vinicius M. de Lima, Bianca B. Batista, José F. da Silva Neto

**Affiliations:** Departamento de Biologia Celular e Molecular e Bioagentes Patogênicos, Faculdade de Medicina de Ribeirão Preto, Universidade de São Paulo, Ribeirão Preto, Brazil

**Keywords:** iron homeostasis, heme uptake, heme transporter, bacterial physiology, bacterial virulence, siderophores, *Chromobacterium violaceum*

## Abstract

*Chromobacterium violaceum* is an environmental Gram-negative beta-proteobacterium that causes systemic infections in humans. *C. violaceum* uses siderophore-based iron acquisition systems to overcome the host-imposed iron limitation, but its capacity to use other iron sources is unknown. In this work, we characterized ChuPRSTUV as a heme utilization system employed by *C. violaceum* to explore an important iron reservoir in mammalian hosts, free heme and hemoproteins. We demonstrate that the *chuPRSTUV* genes comprise a Fur-repressed operon that is expressed under iron limitation. The *chu* operon potentially encodes a small regulatory protein (ChuP), an outer membrane TonB-dependent receptor (ChuR), a heme degradation enzyme (ChuS), and an inner membrane ABC transporter (ChuTUV). Our nutrition growth experiments using *C. violaceum chu* deletion mutants revealed that, with the exception of *chuS*, all genes of the *chu* operon are required for heme and hemoglobin utilization in *C. violaceum*. The mutant strains without *chuP* displayed increased siderophore halos on CAS plate assays. Significantly, we demonstrate that ChuP connects heme and siderophore utilization by acting as a positive regulator of *chuR* and *vbuA*, which encode the TonB-dependent receptors for the uptake of heme (ChuR) and the siderophore viobactin (VbuA). Our data favor a model of ChuP as a heme-binding post-transcriptional regulator. Moreover, our virulence data in a mice model of acute infection demonstrate that *C. violaceum* uses both heme and siderophore for iron acquisition during infection, with a preference for siderophores over the Chu heme utilization system.

## Introduction

Iron is an essential micronutrient required as a cofactor of proteins involved in different cellular processes (Braun and Hantke, 2011; Palmer and Skaar, 2016). The ability to vary from soluble ferrous (Fe^2+^) to insoluble ferric (Fe^3+^) states confers iron its catalytic properties but can result in high toxicity and low bioavailability (Braun and Hantke, 2011; Huang and Wilks, 2017). As a metal essential for both hosts and pathogens, iron is at the center of an evolutionary battle (Skaar, 2010; Hood and Skaar, 2012; Parrow et al., 2013; Sheldon et al., 2016). Hosts restrict iron availability using iron-sequestering proteins like transferrin, lactoferrin, haptoglobin, hemopexin, and calprotectin, a process known as nutritional immunity (Hood and Skaar, 2012; Cassat and Skaar, 2013; Ganz and Nemeth, 2015). Conversely, pathogens subvert the host-imposed iron limitation by employing strategies such as the production, release, and uptake of low-molecular-weight iron chelators (siderophores such as enterobactin) and high-affinity heme-binding proteins (hemophores such as HasA) (Wandersman and Delepelaire, 2012; Runyen-Janecky, 2013; Contreras et al., 2014; Sheldon et al., 2016).

Heme is a tetrapyrrole that coordinates iron at its center as Fe^2+^ (heme) or Fe^3+^ (hemin). It is a cofactor of proteins like cytochromes and catalases. Therefore, almost every organism requires heme, which is obtained by synthesis and/or uptake from exogenous sources (Runyen-Janecky, 2013; Choby and Skaar, 2016). The greatest iron reservoir in mammals is the heme bound into hemoglobin found inside the erythrocytes. Many bacteria use heme and hemoproteins (e.g., hemoglobin) from the host as an iron source, and the preference for heme or siderophore as the main iron acquisition strategy varies according to the bacterium and the infection status (Runyen-Janecky, 2013; Choby and Skaar, 2016; Sheldon et al., 2016; Zygiel et al., 2021). Heme uptake/utilization systems have been described in several bacterial pathogens, including *Pseudomonas aeruginosa* (Has, Phu, and Hxu), *Yersinia* spp (Hem and Hmu), *Escherichia coli* (Chu), and *Staphylococcus aureus* (Isd). In Gram-negative bacteria, the import of heme involves high-affinity TonB-dependent receptors (TBDRs) in the outer membrane (e.g., PhuR) and ABC-type transport systems in the periplasm and inner membrane (e.g., PhuTUV) (Eakanunkul et al., 2005; Noinaj et al., 2010; Fournier et al., 2011; Choby and Skaar, 2016; Huang and Wilks, 2017; Klebba et al., 2021). Once in the cytosol, heme is degraded by canonical or non-canonical heme oxygenases, releasing iron and other compounds (Contreras et al., 2014; Lamattina et al., 2016).

Genes encoding heme uptake systems are under complex regulation. They are regulated by Fur, a metalloregulator that uses Fe^2+^ as cofactor to repress the expression of iron uptake systems (da Silva Neto et al., 2009; Chandrangsu et al., 2017; Sarvan et al., 2018) and activated by heme-dependent regulatory systems, such as the extracytoplasmic function (ECF) sigma factor signaling cascade Has (Wandersman and Delepelaire, 2012; Huang and Wilks, 2017). Small proteins from the HemP/HmuP family have been described as required for heme utilization by regulating the expression of heme uptake genes. However, the proposed regulatory mechanisms are quite distinct. In *Bradyrhizobium japonicum* and *Burkholderia multivorans*, the HemP/HmuP proteins were described as direct transcriptional activators (Escamilla-Hernandez and O’Brian, 2012; Sato et al., 2017), while in *Ensifer meliloti* (formerly *Sinorhizobium meliloti*), HmuP appears to act as a post-transcriptional activator (Amarelle et al., 2010; Amarelle et al., 2019).

*Chromobacterium violaceum* is a Gram-negative beta-proteobacterium found in the water and soil of tropical and subtropical regions that causes opportunistic human infections with high mortality rates (Yang and Li, 2011; Kumar, 2012; Khalifa et al., 2015; Batista and da Silva Neto, 2017). An important virulence determinant in *C. violaceum* is the Cpi1/1a type III secretion system involved in hepatocyte invasion and innate immune system activation (Miki et al., 2010; Zhao et al., 2011; Maltez et al., 2015). Recently, we demonstrated that *C. violaceum* relies on the regulator Fur, two putative endogenous catecholate-type siderophores, and the siderophore-acquisition TBDRs CbuA and VbuA to overcome host-imposed iron limitation (Batista et al., 2019; Santos et al., 2020). However, *C. violaceum* mutants lacking siderophores had moderate attenuation in virulence in a mouse model of acute infection (Batista et al., 2019), suggesting that *C. violaceum* uses siderophore-independent mechanisms for iron acquisition during infection. In the current work, we demonstrate that an operon with six genes, here named *chuPRSTUV* (*chu* – *chromobacterium* heme utilization), encodes a Fur-regulated heme uptake system (ChuRTUV) that is required for heme and hemoglobin utilization in *C. violaceum*. We also show that the small heme-binding protein ChuP is required for heme and siderophore-mediated iron acquisition by acting as a post-transcriptional activator of the TBDR genes *chuR* and *vbuA*. Furthermore, using *in vivo* virulence assays in mice, we demonstrate that these heme and siderophore-mediated iron uptake systems work together to help *C. violaceum* overcome iron limitation in the host.

## Materials and Methods

### Bacterial Strains, Plasmids, and Growth Conditions

The bacterial strains and plasmids used in this work are indicated in **Table 1**. *E. coli* strains were cultured in Luria-Bertani (LB) medium at 37°C. *C. violaceum* strains were cultured in LB medium or M9 minimal medium supplemented with 0.1% casein hydrolysate (M9CH) at 37°C (Batista et al., 2019). The cultures were supplemented with kanamycin (50 μg/mL), tetracycline (10 µg/mL), or ampicillin (100 μg/mL), when necessary. Iron deficiency was obtained by the addition of 2,2’-dipyridyl (DP) (Sigma) to the medium, while iron sufficiency was achieved by supplementation with FeSO_4_ (Sigma), hemin (Hm) (Sigma), or hemoglobin (Hb) (Sigma).

**Table 1:** Bacterial strains and plasmids.

Strain or plasmid	Description^a^	Reference or source
**Strains**		
*E. coli*		
DH5α	*E. coli* strain for cloning purposes	(Hanahan, 1983)
S17-1	*E. coli* strain for plasmid mobilization	(Simon et al., 1983)
BL21(DE3)	*E. coli* strain for heterologous expression of proteins	Novagen
*C. violaceum*		
WT	*C. violaceum* ATCC 12472 wild-type strain with sequenced reference genome	(Brazilian National Genome Project Consortium, 2003)
WT[pMR20]	WT control strain harboring the empty pMR20 plasmid	This work
WT[p*chuP*-*lacZ*]	WT strain with the *chuP* (CV_RS19275)-l*acZ* fusion	This work
WT[p*chuR*-*lacZ*]	WT strain with the *chuR* (CV_RS19280)-l*acZ* fusion	This work
*cbaF*::pNPT	WT strain with insertion of pNPTS138 in the *cbaF* gene	(Batista et al., 2019)
*vbaF*::PNPT	WT strain with insertion of pNPTS138 in the *vbaF* gene	(Batista et al., 2019)
Δ*chuP*	WT strain with the CV_RS19275 gene deleted	This work
Δ*chuP*/*cbaF*::pNPT	Δ*chuP* strain with insertion of pNPTS138 in the *cbaF* gene	This work
Δ*chuP*/*vbaF*::pNPT	Δ*chuP* strain with insertion of pNPTS138 in the *vbaF* gene	This work
Δ*chuP*[*chuP*]	Δ*chuP* mutant complemented with WT copy of *chuP*	This work
Δ*chuP*[p*chuP*-*lacZ*]	Δ*chuP* strain with the *chuP* (CV_RS19275)-l*acZ* fusion	This work
Δ*chuP*[p*chuR*-*lacZ*]	Δ*chuP* strain with the *chuR* (CV_RS19280)-l*acZ* fusion	This work
Δ*chuR*	WT strain with the CV_RS19280 gene deleted	This work
Δ*chuR*[*chuR*]	Δ*chuR* mutant complemented with WT copy of *chuR*	This work
Δ*chuS*	WT strain with the CV_RS19285 gene deleted	This work
Δ*chuS*[*chuS*]	Δ*chuS* mutant complemented with WT copy of *chuS*	This work
Δ*chuTUV*	WT strain with the CV_RS19290-295-300 genes deleted	This work
Δ*chuTUV*[*chuTUV*]	Δ*chuTUV* mutant complemented with WT copy of *chuTUV*	This work
Δ*chuPRSTUV*	WT strain with the CV_RS19275-280-285-290-295-300 genes deleted	This work
Δ*chuPRSTUV* [*chuPRSTUV*]	Δ*chuPRSTUV* mutant complemented with WT copy of *chuPRSTUV*	This work
Δ*cbaCEBA*	WT strain with the *cbaCEBA* genes deleted	(Batista et al., 2019)
Δ*cbaCEBA*[*cbaCEBA*]	Δ*cbaCEBA* mutant complemented with WT copy of *cbaCEBA*	(Batista et al., 2019)
Δ*cbaCEBA*Δ*chuPRSTUV*	WT strain with combined mutations of *cbaCEBA* and *chuPRSTUV*	This work
Δ*cbaCEBA*Δ*chuPRSTUV*[pMR20]	Δ*cbaCEBA*Δ*chuPRSTUV* mutant harboring the empty pMR20 plasmid	This work
*ΔcbaCEBA∆chuPRSTUV*[*chuPRSTV*]	Δ*cbaCEBA*Δ*chuPRSTUV* mutant complemented with WT copy of *chuPRSTUV*	This work
Δ*fur*	WT strain with the *fur* gene deleted	(Santos et al., 2020)
Δ*fur*[p*chuP*-*lacZ*]	Δ*fur* strain with the *chuP* (CV_RS19275)-l*acZ* fusion	This work
**Plasmids**		
pNPTS138	Suicide vector containing *oriT*, *sacB*; Kan^R^	M.R.K. Alley
pMR20	Broad-host-range low-copy vector containing *oriT*, Tet^R^	(Roberts et al., 1996)
pET15b	Expression of proteins with N-terminal His-tag; Amp^R^	Novagen
pGEM-T easy	Cloning plasmid; Amp^R^	Promega
pRK*lacZ*290	pRK2-derived vector with promoterless *lacZ* gene, Tet^R^	(Gober and Shapiro, 1992)

a Kan, kanamycin; Tet, tetracycline; Amp, ampicillin; R, resistance.

### Construction of *C. violaceum* Mutant and Complemented Strains

Null-mutant strains were generated by a previously established allelic exchange mutagenesis protocol (da Silva Neto et al., 2012; Batista et al., 2019; Santos et al., 2020). In-frame null-deletion mutants derived from the wild-type *C. violaceum* ATCC 12472 strain, with the exception of the Δ*cbaCEBA*Δ*chuPRSTUV* strain that was obtained using the Δ*cbaCEBA* mutant as background (Batista et al., 2019). The insertion mutants for the non-ribosomal peptide synthetase (NRPS) genes *cbaF* and *vbaF* were obtained by a protocol based on a single recombination event (Batista et al., 2019). For genetic complementation, the *chuP*, *chuR*, *chuS*, *chuTUV*, and *chuPRSTUV* genes were amplified by PCR, cloned into the low-copy-number plasmid pMR20, and transferred to the mutant strains by conjugation. The primers used for cloning, sequencing, and mutant confirmation are listed in **Supplementary Table 1**.

### MIC Assay

To achieve iron-limited conditions in M9CH for *C. violaceum*, we determined the minimal inhibitory concentration (MIC) of DP in this medium, as previously performed in LB medium (Batista et al., 2019). Wild-type *C. violaceum* overnight cultures were diluted to an optical density at 600 nm (OD_600_) of 0.01 in M9CH, without or with DP (100 µM, 112.5 µM, 125 µM, 132.5 µM, and 150 µM), and grown under agitation (250 rpm) at 37°C. The MIC of 132.5 µM DP for the WT strain was established based on the turbidity of the cultures after 24 h cultivation.

### Growth Curves

Growth curves were determined in M9CH without or with Hm. Overnight cultures of *C. violaceum* strains were diluted in 5 mL of M9CH to an OD_600_ of 0.02. Then, a new dilution (1:2) was performed to achieve an OD_600_ 0.01 and the required concentrations of Hm in 200 µL final M9CH in 96-well plates. The plates were incubated at 37°C under moderate orbital agitation in SpectraMax i3 MiniMax Imaging Cytometer (Molecular Devices). The measurements of OD_600_ were recorded every 15 minutes over 24 hours. The experiment was performed in three biological replicates.

### Heme and Hemoglobin Nutrition Assay

The ability of *C. violaceum* to use Hm and Hb as iron sources was assessed using a nutrition assay (Balhesteros et al., 2017) with some modifications. *C. violaceum* overnight cultures in M9CH were diluted to an OD_600_ of 1.0 in M9CH. Then, 25 µL of each dilution were embedded in 25 mL of iron-depleted M9CH 0.8% agar (containing 50 µM, 100 µM, 125 µM or 150 µM of DP). Paper discs were added onto the plate surface, and 10 µL aliquots of 100 µM Hm, 20 mM NaOH, 150 µM Hb, and 100 mM NaCl were applied to individual discs. After incubation for 16 h at 37°C, we inspected for growth halos that developed around the discs. The growth area was quantified using the Image J software and normalized by subtracting the disc areas. The experiment was performed in three biological replicates.

### Cell Viability in the Presence of Heme

The toxic concentrations of Hm for *C. violaceum* were assessed by cell viability. Overnight cultures were diluted to an OD_600_ of 0.01 in M9CH without or with Hm (30 µM, 600 µM, and 2000 µM), and grown under agitation (250 rpm) for 24 h at 37°C. Serial dilution in phosphate-buffered saline (PBS) was performed, and 10 µL were spotted onto M9CH plates. Hemin toxicity was determined based on the colony-forming units (CFU) displayed by the strains after incubation for 24 h at 37°C. These experiments were performed in three biological replicates.

### Hemolysis Assay

The hemolytic activity was assessed in 5% (v/v) sheep-blood Mueller-Hinton agar plates. Five microliters of *C. violaceum* M9CH overnight cultures were spotted onto the plate. The hemolytic activity was detected by the lighter halos that developed due to erythrocyte lysis after incubation for 7 days at 37°C. The area of activity was quantified using the Image J software and normalized by subtracting the bacterial growth area. The experiment was performed in three biological replicates.

### Siderophore Assay

Siderophores were detected by chrome azurol S (CAS) plate assay in modified peptone-sucrose agar (PSA-CAS) plates (Batista et al., 2019; Santos et al., 2020). Ten microliters of *C. violaceum* overnight cultures in M9CH were spotted onto the plate surface, and the siderophores were detected by the orange halos that developed after incubation for 24 hours at 37°C. The area of the halos was quantified using the Image J software and normalized by subtracting the bacterial growth area. The experiment was performed in three biological replicates.

### Transcriptional *lacZ* Fusions and β-Galactosidase Assays

The upstream regions of genes of interest were amplified by PCR with specific primers (**Supplementary Table 1**) and cloned into the pGEM-T easy plasmid (Promega). After digestion with proper restriction enzymes (**Supplementary Table 1**), the inserts were subcloned into the pRKl*acZ*290 vector to generate transcriptional fusions to the *lacZ* gene. *C. violaceum* cultures harboring the reporter plasmids were grown until an OD_600_ of 0.6 – 0.8 in M9CH, and either untreated or treated with 100 µM Hm or 100 µM FeSO_4_ for 2 h. For all expression assays, the M9CH medium was used as the iron-limited condition because we previously found that the expression of an iron-regulated gene was similarly high in M9CH or M9CH with DP (Santos et al., 2020). Bacterial cells were assayed for β-galactosidase activity as previously described (Santos et al., 2020). The experiment was performed in three biological replicates.

### Co-Transcription by RT-PCR

The *C. violaceum* wild-type strain was grown in M9CH until an OD_600_ of 1.0 – 1.2. Total RNA was extracted using Trizol reagent (Invitrogen) and purified with Direct-zol™ RNA Miniprep Plus (Zymo Research). RT-PCR was performed with the SuperScript III One-Step RT-PCR System with Platinum Taq High Fidelity DNA Polymerase (Invitrogen). One microgram of each RNA sample and specific primers (**Supplementary Table 1**) that amplify regions from *chuP* to *chuR* (439 bp), *chuR* to *chuS* (373 bp), and *chuS* to *chuT* (662 bp) were used in the reactions. PCRs using conventional Taq DNA polymerase, and the same sets of primers, were performed with genomic DNA (positive control) and RNA (negative control) as templates.

### Gene Expression by RT-qPCR

The *C. violaceum* wild type, Δ*chuP*, and Δ*chuP*[*chuP*] strains were grown in M9CH until midlog growth phase, and the cultures were either untreated or treated with 100 µM Hm or 100 µM FeSO_4_ for 2 h. Total RNA was extracted and purified as described above. Two micrograms of total RNA from each sample were converted to cDNA using the High-Capacity cDNA Reverse Transcription kit (Thermo Fisher Scientific). Genomic DNA contamination (for RNA) and reverse transcription efficiency (for cDNA) were checked by conventional PCR with the primers for the *rpoH* gene (**Supplementary Table 1**). Quantitative PCR (qPCR) reactions were performed using the PowerUp™ SYBR™ Green Master Mix (Thermo Fisher Scientific), the specific primers (**Supplementary Table 1**), and 0.5 µL of cDNA. The relative expression was calculated by the 2^-ΔΔCt^ method (Livak and Schmittgen, 2001). Data from three biological replicates were normalized by an endogenous control (*rpoH* gene) and a reference condition (WT in M9CH 100 µM Hm). The treatment with Hm was used as a control based on the β-galactosidase assays that indicated an intermediate expression of the *chu* operon under this condition.

### Expression and Purification of the Recombinant ChuP

The coding region of *chuP* was amplified by PCR (**Supplementary Table 1**) and cloned into the pET-15b plasmid (**Table 1**). After induction in *E. coli* BL21(DE3) with 1 mM Isopropyl β-D-1-thiogalactopyranoside (IPTG) for 2 h at 37°C, the His-ChuP protein was purified from the soluble extract by affinity chromatography in a Ni-NTA Superflow column (Qiagen). The elution fractions were evaluated using 18% SDS-PAGE. The aliquots containing the purified His-ChuP were concentrated using a VivaSpin 6 column (Sartorius), and desalted by gel filtration in PD-10 column (GE Healthcare) in storage buffer (100 mM NaH_2_PO_4_, 600 mM NaCl, 20% glycerol, pH 8) (Puri and O’Brian, 2006). The concentration of the His-ChuP protein was determined by measurement of OD at 280 nm and using its extinction coefficient calculated by the Protparam Tool (ExPASy) (http://web.expasy.org/protparam).

### Heme Binding Assay

The ability of the recombinant His-ChuP protein to interact with Hm was evaluated by spectrophotometry (Puri and O’Brian, 2006; Amarelle et al., 2016). The reactions were performed in interaction buffer (50 mM Na_2_H_2_PO_4_, 300 mM NaCl, 10% glycerol, pH 8) without (reference cuvette) or with 10 µM of His-ChuP (sample cuvette). Aliquots of Hm (0 to 30 µM) were added to both cuvettes. After incubation for 5 minutes at 25°C in the dark, the absorbance between the wavelengths 300 and 600 nm was measured with 10 nm increments on a SpectraMax i3 MiniMax Imaging Cytometer. The binding of ChuP to Hm was determined by the change in absorbance at 413 nm fit to one-site binding model non-linear regression on Graph Pad Prism 7.

### Electrophoretic Mobility Shift Assay (EMSA)

DNA sequences upstream of *chuP*, *chuR*, and CV_2599 were amplified by PCR using the primers listed in **Supplementary Table 1**. The DNA fragments were radiolabeled and used for interaction with His-ChuP following a previously described protocol (da Silva Neto et al., 2009; Previato-Mello et al., 2017), with the modification of adding the CV_2599 promoter fragment (negative control) in the same reaction.

### Mouse Virulence Assays

Virulence assays were performed in a mouse intraperitoneal (i.p.) model of *C. violaceum* infection as previously established (Previato-Mello et al., 2017; Batista et al., 2019). Bacterial strains were diluted to an OD_600_ of 0.01 and cultured in 5 mL LB for 20 h at 37°C. A dose of 10^6^ CFU in PBS was injected into 6-week-old female BALB/c mice, and the animals were monitored for 7 days post-infection. To assess the bacterial burden in the liver and spleen, mice were infected as above and euthanized 20 h or 96 h post-infection (h.p.i.). The organs were aseptically collected, homogenized in PBS, and the dilutions were plated for CFU counting. Mice were obtained and maintained at the Animal Facilities of Ribeirão Preto Medical School (FMRP-USP). The assays were performed according to the Ethical Principles in Animal Research adopted by the National Council for the Control of Animal Experimentation (CONCEA). The animal ethics protocol 146/2019 was approved by the Local Ethics Animal Committee (CEUA) of FMRP-USP.

### Statistical Analysis

Data collected were employed for statistical analysis in GraphPad Prism version 7. For the column graphs, the normality test was performed using Shapiro-Wilk’s test. Statistically significant p values and the tests that were performed are indicated in the figure legends.

## Results

### The *chuPRSTUV* Operon Is Regulated by Fur According to the Iron Levels

**Figure 1:**
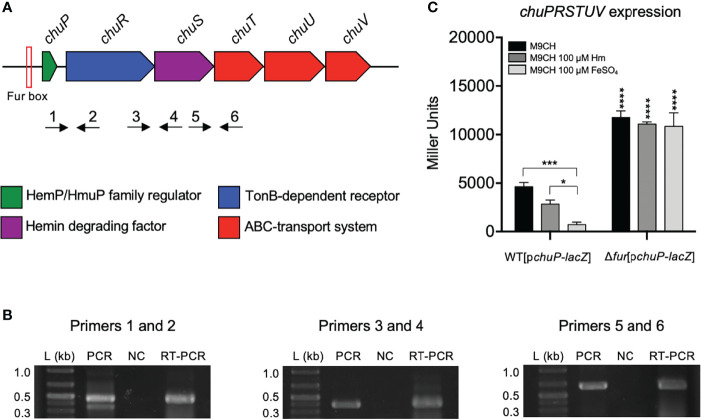
The *chuPRSTUV* genes compose an operon regulated by the iron levels and Fur. **(A)** Genomic organization of the *chuPRSTUV* genes in *C. violaceum*. A predicted Fur box is indicated. Numbered arrows indicate the primers used in RT-PCR (not scaled). **(B)** Confirmation of co-transcription of the *chuPRSTUV* genes. The RT-PCR reactions amplified fragments of 439 bp (Primers 1 and 2), 373 bp (Primers 3 and 4), and 662 bp (Primers 5 and 6). Conventional PCR was performed using genomic DNA (PCR) and RNA (NC) as controls. L, 1 Kb plus DNA Ladder (Thermo Scientific). **(C)** Promoter activity of the *chu* operon in response to iron and Fur. β-galactosidase assays were performed from the WT and Δ*fur* strains harboring the *chuP-lacZ* fusion grown in M9CH medium and either untreated or treated with 100 μM Hm or 100 μM FeSO_4_. Data are from three biological replicates. ****p < 0.0001; ***p < 0.001; *p < 0.05; when not indicated, not significant. Two-way ANOVA followed by Dunnett’s multiple-comparison test.

*In silico* analysis of the *C. violaceum* ATCC 12472 genome sequence revealed a gene cluster with six genes (CV_RS19275-280-285-290-295-300) that resembles an operon encoding a putative heme utilization system. These genes, here named *chuPRSTUV*, are annotated as a HemP/HmuP family regulator (ChuP), a TonB-dependent receptor (ChuR), a hemin degrading factor (ChuS), and an ABC-transport system (ChuTUV) (**Figure 1A**). To evaluate if the *chuPRSTUV* genes are organized into an operon, we performed RT-PCR reactions using RNA from the WT strain grown in M9CH and a set of primers that amplify regions between *chuPR*, *chuRS*, and *chuST* genes (**Figures 1A, B**). After reverse transcription and amplification, bands with the expected sizes were detected for the three tested primer combinations, confirming that the *chuPRSTUV* genes are indeed co-transcribed (**Figure 1B**).

Our inspection of the promoter region of *chuP* revealed a putative Fur binding site sequence (ATGATAATGGTTATCATT) that resembles Fur boxes found in other bacteria (Sarvan et al., 2018). To investigate whether the *chu* operon is regulated by iron and Fur, we cloned the promoter region of *chuP* into a *lacZ* reporter plasmid. The WT and Δ*fur* strains harboring the p*chuP*-*lacZ* fusion were used to assess the *chuP* promoter activity by β-galactosidase assay in M9CH medium, which was previously reported as an iron-limited condition (Santos et al., 2020) and under iron sufficiency (M9CH supplemented with Hm or FeSO_4_) (**Figure 1C**). The promoter activity was higher under iron-limited (M9CH) than iron-replete conditions in the WT strain. The reduction in activity was higher with FeSO_4_ than with Hm supplementation. In the Δ*fur* mutant, the promoter was highly active regardless of iron levels. Moreover, the activity was twofold higher than that detected for the WT strain in M9CH, suggesting total promoter de-repression in the absence of Fur (**Figure 1C**). Altogether, these results demonstrate that the *chuPRSTUV* genes comprise a Fur-repressed operon that is expressed under iron limitation.

### The *chuPRSTUV* Operon Encodes a Heme Uptake System (ChuRTUV) and a Regulatory Protein (ChuP) Required for Heme and Hemoglobin Utilization

To characterize the role of the *chuPRSTUV* operon in *C. violaceum*, we generated null-mutant strains deleted for single genes (Δ*chuP*, Δ*chuR*, and Δ*chuS*) or multiple genes (Δ*chuTUV* and Δ*chuPRSTUV*) of the *chu* operon, and their respective complemented strains. We also obtained a mutant strain lacking both the *chu* operon and the *cbaCEBA* genes (Δ*cbaCEBA*Δ*chuPRSTUV*). The CbaCEBA enzymes are involved in the synthesis of 2’3-DHB, the precursor of catecholate-type siderophores in *C. violaceum* (Batista et al., 2019). All mutants showed regular fitness, as assessed by growth curves in M9CH and M9CH plus heme and by cell viability in LB (**Supplementary Figure 1**).

**Figure 2:**
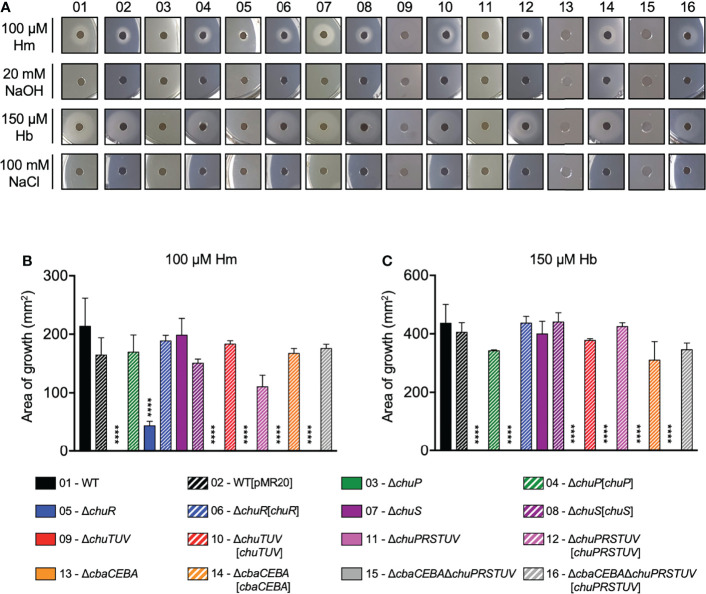
The *chu* operon encodes a heme uptake system (ChuRTUV) and a regulatory protein (ChuP) required for heme and hemoglobin utilization. **(A)** Nutrition assay for Hm and Hb under DP-imposed iron deficiency. The indicated strains were embedded into M9CH medium supplemented with 125 μM DP. Aliquots of 100 μM Hm and 150 μM Hb were provided as iron sources, while 20 mM NaOH and 100 mM NaCl were used as negative controls. Growth halos around the discs indicate compound utilization. Representative images are shown. **(B, C)** Quantification of Hm and Hb-stimulated growth. The area of the growth halos stimulated by Hm **(B)** and Hb **(C)** was measured using Image J software by subtracting the area of the discs. Data are from three biological replicates. Mutant and complemented strains were compared to WT and WT[pMR20], respectively. ****p < 0.0001; when not indicated, not significant. One-way ANOVA followed by Tukey’s multiple-comparison test.

To test the involvement of the *C. violaceum chu* genes in heme and hemoglobin utilization, we developed a nutrition assay providing 100 µM Hm or 150 µM Hb as alternative iron sources in M9CH medium chelated for iron with different DP concentrations (**Supplementary Figure 2**). We chose 125 µM DP to compare all strains (**Figure 2**) because it was the best condition to visualize the growth halos in the WT strain (**Supplementary Figure 2**). Under these conditions (125 µM DP), the WT and the Δ*chuS* strains formed Hm and Hb-stimulated growth halos. All the other mutant strains of the *chuPRSTUV* operon lost the ability to grow when Hm and Hb were provided as iron sources (**Figure 2**). For the Δ*chuR* strain, a very weak growth stimulus could still be detected only in the presence of heme (**Figure 2B**). Genetic complementation of the mutant strains fully restored the growth in Hm and Hb under deficiency with 125 µM DP (**Figure 2**). The Δ*cbaCEBA* mutant that does not synthesize siderophores showed no growth halos at 125 µM DP (**Figure 2**), but its growth was clearly stimulated by Hm and Hb at 50 µM DP (**Supplementary Figure 2**). This is consistent with previous results indicating that the growth of a Δ*cbaCEBA* mutant is strongly impaired under DP-imposed iron limitation (Batista et al., 2019). Taken together, these results demonstrate that the *chuPRSTUV* operon encodes a heme uptake system (ChuRTUV) that is also involved in hemoglobin utilization. Moreover, the weak growth detected for the Δ*chuR* mutant with Hm but not with Hb suggests that *C. violaceum* has additional mechanisms in the outer membrane for heme uptake but relies specifically on ChuR for heme uptake from hemoglobin.

Considering that the Δ*chuS* strain showed no altered phenotype for Hm and Hb utilization (**Figure 2**), we evaluated its role on cell viability under heme excess (**Supplementary Figure 3**). However, growth defects were not observed for the WT, Δ*chuS*, and all mutant strains even at a high Hm concentration of 2 mM, indicating that the *chu* operon has no role during our heme excess conditions. Interestingly, deletion of the *chuPRSTUV* operon in the Δ*cbaCEBA* mutant strain improved the small colony size phenotype (**Supplementary Figure 3**), previously described for this strain (Batista et al., 2019). We also tested the hemolytic activity of the *chu* mutants on sheep-blood agar (**Supplementary Figure 4**). The strains Δ*chuR* (increased and intense halo) and Δ*cbaCEBA* (intense halo) showed altered hemolytic activity when compared to that of the WT and the other mutant strains. Although the meaning of these findings is unclear, we speculate that the increased hemolytic activity in these strains is a compensatory mechanism to deal with iron/heme scarcity.

### The Δ*chuP* Mutant Has Increased Siderophore Halos Due to Viobactin

**Figure 3:**
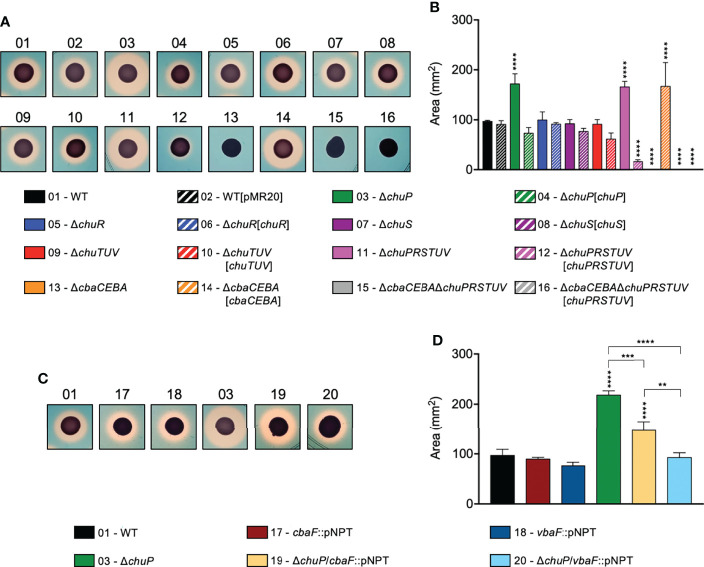
Deletion of *chuP* impacts the siderophore levels in *C. violaceum*. **(A, B)** Role of the *chu* operon on the siderophore levels in *C. violaceum*. Mutant strains without *chuP* showed increased siderophore halos. **(C, D)** The effect of ChuP occurs on the siderophore viobactin. For all indicated strains, the siderophore detection was performed by CAS assays on PSA-CAS plates. *C. violaceum* cultures were spotted onto the plate surface, and the orange halos indicating secreted siderophores were photographed **(A, C)** and measured **(B, D)**, after incubation for 24 hours at 37°C, using Image J software. The area of the siderophore halos was calculated subtracting the area of bacterial growth. Data are from three biological replicates. Mutant and complemented strains **(B)** were compared to WT and WT[pMR20], respectively. Insertion mutants **(D)** were compared to the strains they derived from. **p < 0.01; ***p < 0.001; ****p < 0.0001; when not indicated, not significant. Vertical asterisks indicate comparisons with the WT strain. One-way ANOVA followed by Tukey’s multiple-comparison test.

To verify whether the *chu* operon affects the production/release of siderophores in *C. violaceum*, we tested the *chu* mutants on PSA-CAS plates for siderophore detection as orange halos (**Figure 3**) as previously described (Batista et al., 2019). The Δ*chuP* and Δ*chuPRSTUV* mutants showed increased siderophore halos, while the Δ*chuR*, Δ*chuS*, and Δ*chuTUV* mutants had siderophore halos similar to that of the WT strain (**Figures 3A, B**). The Δ*cbaCEBA* strain showed no siderophore halo, as previously demonstrated (Batista et al., 2019), as well as the Δ*cbaCEBA*Δ*chuPRSTUV* strain (**Figures 3A, B**). After complementation, the siderophore halos were restored to WT levels in the Δ*chuP*[*chuP*] strain. For the strains Δ*chuPRSTUV*[*chuPRSTUV*] (almost absence of halo) and Δ*cbaCEBA*[*cbaCEBA*] (increased halo), the siderophore phenotypes reverted further on that observed in the WT strain (**Figures 3A, B**), perhaps owing to overexpression of the genes into the plasmid. These data indicate that the small regulatory protein ChuP controls the siderophore levels in *C. violaceum*.

*C. violaceum* produces the catecholate-type siderophores chromobactin and viobactin employing the NRPS enzymes CbaF and VbaF, respectively (Batista et al., 2019). We combined mutation in *chuP* with mutations in *cbaF* or *vbaF* to understand which siderophore contributes to the increased siderophore halos in Δ*chuP*. Individual deletion of *cbaF* or *vbaF* genes in the WT had no effect on siderophore halos (**Figures 3C, D**), as previously reported (Batista et al., 2019). When these genes were deleted in the Δ*chuP* mutant background, a decrease in siderophore halos was observed in both cases. However, the halos were similar to that of the WT strain only when *vbaF* was deleted (**Figures 3C, D**), demonstrating a prominent role of viobactin on the increased siderophore halos of Δ*chuP*. Altogether, these results suggest that ChuP controls the synthesis and/or uptake of the siderophore viobactin in *C. violaceum*.

### ChuP Is a Heme-Binding Post-Transcriptional Regulator of *chuR* and *vbuA* Encoding TBDRs for Heme and the Siderophore Viobactin

**Figure 4:**
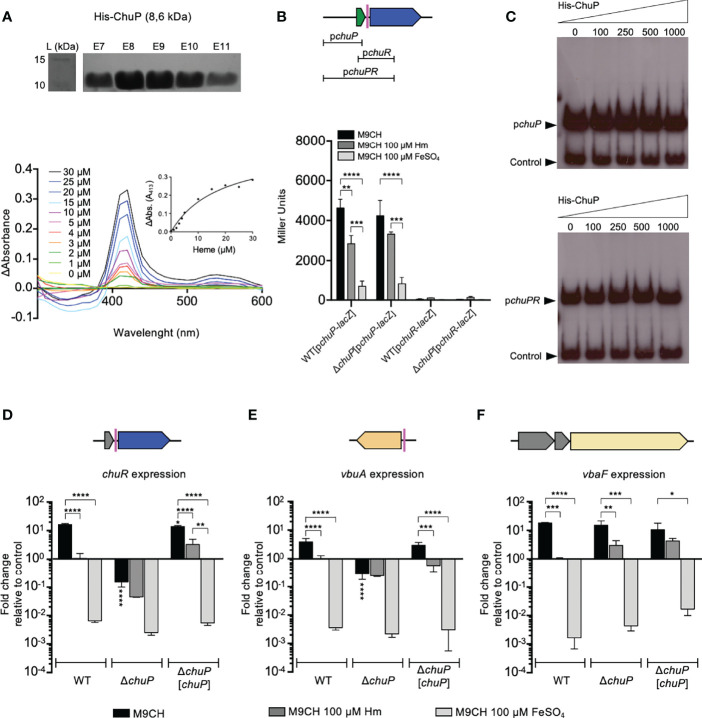
ChuP is a heme-binding regulatory protein that controls *chuR* and *vbuA* expression at a post-transcriptional level. **(A)** ChuP binds heme. The His-ChuP protein was purified (top) and incubated (10 μM protein) with the indicated concentrations of Hm (bottom). The absorption spectra were measured from 300 nm to 600 nm on a SpectraMax i3 MiniMax Imaging Cytometer. The changes at 413 nm were used to calculate the ChuP-Hm affinity (insert). Data are shown as differential absorption spectra: the difference of values obtained from the sample cuvette (His-ChuP and Hm) against the reference cuvette (Hm). Data are from a single experiment of three independent replicates. L, protein ladder; E7 to E11, eluted fractions of purified ChuP. **(B)** ChuP does not regulate the promoter of the *chu* operon. The scheme (top) indicates the regions used for β-galactosidase or EMSA assays. β-galactosidase assays were performed from the WT and Δ*chuP* strains harboring *chuP-lacZ* and *chuR*-*lacZ* fusions grown in M9CH in the indicated conditions of iron availability. Data are from three biological replicates. **p < 0.01; ***p < 0.001; ****p < 0.0001. When not shown, n.s. (not significant). Two-way ANOVA followed by Dunnett’s multiple-comparison test. **(C)** ChuP does not bind to DNA probes covering from *chuP* to *chuR*. The indicated concentrations of His-ChuP were used in EMSA assays with the *chu* indicated probes. In both cases, the promoter region of CV_2599 (control) was used as an in-reaction unspecific negative control. **(D-F)** *chuR* and *vbuA* but not *vbaF* have HPRE sequences and are regulated by ChuP. The predicted HPREs are indicated as colored bars in the gene maps. Expression was evaluated by RT-qPCR. cDNA was reverse transcribed from RNA obtained from the WT, Δ*chuP*, and Δ*chuP*[*chuP*] strains grown in M9CH, and either untreated or treated with 100 μM Hm or 100 μM FeSO_4_. Expression of *chuR*, *vbuA*, and *vbaF* is shown as the fold change relative to the control condition (WT in M9CH 100 μM Hm). Data are from three biological replicates. ****p < 0.0001; ***p < 0.001; **p < 0.01; *p < 0.05; when not indicated, not significant. Vertical asterisks indicate comparisons with the WT strain at the same condition. One-way ANOVA followed by Tukey’s multiple-comparison test.

Our data indicate that mutation of *chuP* in *C. violaceum* abolished heme utilization (**Figure 2**) and altered the levels of the siderophore viobactin (**Figure 3**). We employed different methodologies to elucidate how ChuP regulates these processes (**Figure 4**). First, we tested whether ChuP is a heme-binding protein. We purified the recombinant protein His-ChuP and performed a heme-binding assay (**Figure 4A**). After incubation with increasing Hm concentrations, a Soret peak at 413 nm was detected, indicating the formation of a ChuP-heme complex (**Figure 4A**). The differential absorption spectroscopy at 413 was used to fit a single binding model and determined that ChuP binds heme with a k_d_ = 18.36 ± 4.66 µM (**Figure 4A**, insert). Considering that HemP/HmuP proteins have been described as transcriptional activators (Escamilla-Hernandez and O’Brian, 2012; Sato et al., 2017), we tested whether the *C. violaceum* ChuP regulates and binds into the intergenic regions upstream of *chuP* (promoter of the *chu* operon) and *chuR* (**Figures 4B, C**). The WT and Δ*chuP* strains harboring these constructs (p*chuP*-*lacZ* or p*chuR*-*lacZ*) were assessed by β-galactosidase assay under different iron levels. The p*chuP*-*lacZ* promoter fusion was highly active under iron deficiency (M9CH) with a gradual decrease upon Hm and FeSO_4_ supplementation (**Figure 4B**), as previously observed in the WT strain (**Figure 1C**). However, the same activity pattern was detected in the Δ*chuP* mutant strain, indicating that ChuP does not seem to regulate the promoter of the *chu* operon (**Figure 4B**). The p*chuR*-l*acZ* fusion had no promoter activity regardless of the strain or condition, indicating the absence of a promoter upstream of *chuR*. Therefore, this fusion is not useful to verify the effect of ChuP on *chuR* expression. Consistent with the β-galactosidase assays, our EMSA assays indicated that ChuP does not bind to the probes containing only the promoter of the *chu* operon or containing the entire region from *chuP* to *chuR* (**Figure 4C**). Altogether, these results demonstrate that ChuP does not regulate the promoter of the *chu* operon nor act as a DNA binding protein.

In *E. meliloti*, the HmuP protein activates the expression of the TBDR ShmR at a post-transcriptional level, probably by acting on a sequence HPRE (HmuP-responsive element). The HPRE sequences were predicted upstream of genes encoding heme TBDRs in many bacteria, including the *C. violaceum chuR* (sequence CCCGCAAGCCAGCCGACAGCCAGCCAGCG, -26 nt from the ATG start codon) (Amarelle et al., 2019). In addition to *chuR*, we found an HPRE sequence upstream of *vbuA* (sequence GCCAGCCAGACGACGCCGCCG, -49 nt from the ATG start codon), a TBDR gene located far from the *chu* operon (**Figures 4D, E**), raising the possibility that ChuP is a post-transcriptional regulator in *C. violaceum*. To verify this hypothesis, we performed RT-qPCR for *chuR*, *vbuA*, and *vbaF* genes with RNA harvested from the WT, Δ*chuP*, and Δ*chuP*[*chuP*] under different iron levels (**Figures 4D–F**). The expression of the three genes in the WT strain was high under iron-depleted and low under iron-sufficient conditions when compared to the control condition (WT grown in M9CH 100 µM Hm), as expected for genes related to iron acquisition (**Figures 4D–F**). Consistent with our phenotypic results and the presence of HPRE elements, the expression of *chuR* and *vbuA* was decreased in the Δ*chuP* strain regardless of the iron levels (**Figures 4D, E**), indicating that ChuP is a positive regulator required for the maximum expression of *chuR* and *vbuA* under iron limitation. Complementation of Δ*chuP* restored the expression of *chuR* and *vbuA* to the levels found in the WT strain (**Figures 4D, E**). No differences in *vbaF* expression were detected between the WT and Δ*chuP* strains in any of the tested conditions (**Figure 4F**), indicating that ChuP does not control the expression of VbaF, the NRPS for viobactin synthesis. Therefore, the decreased expression of *chuR* and *vbuA* in Δ*chuP* explains the inability of this mutant strain to use Hm and Hb (**Figure 2**) (via ChuR) and its increased siderophore halos (**Figure 3**) (inability to uptake viobactin *via* VbuA). Indeed, a Δ*vbuA* mutant showed large siderophore halos (Batista et al., 2019) as those found in Δ*chuP*. Altogether, these results demonstrate that ChuP integrates the acquisition of heme and siderophore by acting as a heme-binding post-transcriptional regulator of the TBDR genes *chuR* and *vbuA*.

### *C. violaceum* Employs Both Siderophores and Heme for Iron Acquisition During Infection

**Figure 5:**
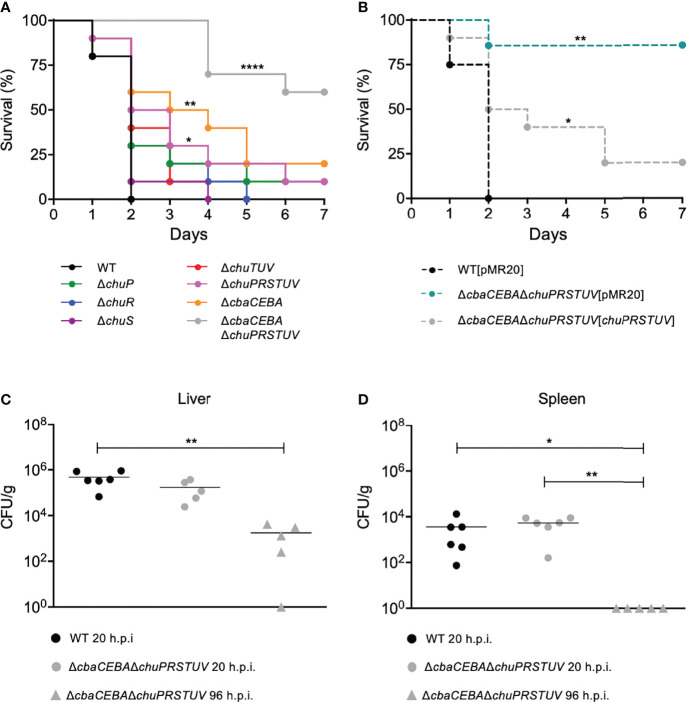
*C violaceum* requires siderophores and heme but prefers siderophores in a mice model of acute infection. **(A, B)** Survival curves of infected BALB/c mice. Animals (n = 8 for WT[pMR20]; n = 7 for Δ*cbaCEBA*Δ*chuPSRTUV*[*chuPRSTUV*]; n = 10 for all other strains) were i.p. injected with 10^6^ CFU of the indicated *C. violaceum* mutant **(A)** and complemented **(B)** strains. Animal survival was monitored daily for a week. *p < 0.05; **p < 0.01; ****p < 0.0001; when not shown, n.s. (not significant). Log-rank (Mantel-Cox) test. **(C, D)** Bacterial burden in organs. Animals were infected with 10^6^ CFU of the indicated strains. After 20h or 96 h post-infection (h.p.i.), the liver **(C)** and the spleen **(D)** were collected, homogenized, serially diluted, and plated for CFU quantification. *p < 0.05; **p < 0.01; when not indicated, not significant. One-way ANOVA followed by Tukey’s (liver) or Dunnett’s (spleen) multiple-comparison tests.

To assess the role of the heme utilization system ChuPRSTUV during *C. violaceum* infection, we performed mice virulence assays (**Figure 5**). The animals were i.p. injected with a dose of 10^6^ bacterial cells and analyzed for survival during seven days post-infection (**Figures 5A, B**). The five null-mutant strains of the ChuPRSTUV system showed barely or no virulence attenuation compared to the *C. violaceum* WT strain (**Figure 5A**). Previously, we determined that abrogating siderophore production in *C. violaceum* by deletion of the *cbaCEBA* genes causes moderate attenuation in virulence (Batista et al., 2019). Therefore, we checked whether heme and siderophores cooperate for virulence. Indeed, the Δ*cbaCEBA* strain showed an intermediate virulence attenuation, as expected, while a more expressive virulence attenuation was observed for the Δ*cbaCEBA*Δ*chuPRSTUV* strain (**Figure 5A**). Complementation of the latter strain with the *chuPRSTUV* operon reverted its virulence attenuation phenotype to the pattern observed for the Δ*cbaCEBA* mutant (**Figure 5B**).

We evaluated the bacterial burden in the liver and spleen, two organs colonized during *C. violaceum* infection that are involved in host heme recycling. Interestingly, the Δ*cbaCEBA*Δ*chuPRSTUV* mutant displayed the same CFU counting as the WT strain at 20 hours post-infection in both organs (**Figures 5C, D**). However, the bacterial burden was reduced (in the liver) and eliminated (in the spleen) at 96 hours post-infection (**Figures 5C, D**). These results indicate that the absence of siderophores and heme uptake does not impair initial colonization but impairs the bacterial maintenance in later infection stages. Altogether, these results indicate an interplay between the iron-acquisition strategies based on siderophore and heme during *C. violaceum* infection. In our acute infection model, the requirement of heme uptake for virulence becomes evident in the absence of siderophores.

## Discussion

**Figure 6:**
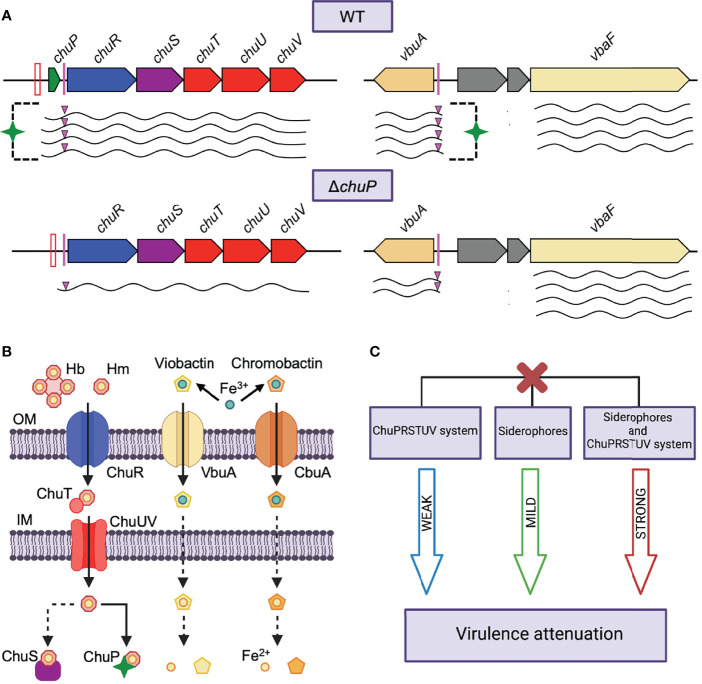
Model of how *C. violaceum* connects iron acquisition by heme and siderophore during infection. **(A)** Regulatory function of ChuP over *chuR* and *vbuA*. **(B)** Iron acquisition systems in *C. violaceum* for the uptake of heme (this work) and the siderophores viobactin and chromobactin (Batista et al., 2019). **(C)** Interplay between the uptake of heme and siderophore in the *C. violaceum* virulence. We propose that ChuP links heme and siderophore utilization by acting as a positive regulator of *chuR* and *vbuA*, which encode TBDRs for the uptake of heme (ChuR) and the siderophore viobactin (VbuA). In addition to Fur derepression, the expression of *chuR* and *vbuA* depends on ChuP under iron deficiency, possibly by a post-transcriptional mechanism involving HPRE (HmuP-responsive elements) sequences found in the 5’-UTR of the *chuR* and *vbuA* transcripts. In the absence of ChuP, the abundance of *chuR* and *vbuA* transcripts decreases, causing a reduction in heme and viobactin utilization. Different levels of virulence attenuation occurred when the ChuPRSTUV system (weak), the siderophores (mild), or both (strong) were deleted, indicating that *C. violaceum* prefers siderophores over heme during infection but relies on heme in the absence of siderophores. Red bars, Fur boxes; Pink bars and triangles, predicted HPRE sequences; Green stars, ChuP protein; Waved lines, mRNAs; Red X, mutant strains; OM, Outer membrane; IM, Inner membrane; Dashed lines, unknown mechanisms.

In this work, we identified and characterized a heme and hemoglobin utilization system, here named ChuPRSTUV, which connects, *via* the regulatory protein ChuP, iron acquisition by heme and siderophore during *C. violaceum* infection (**Figure 6**). Our data indicated that the genes *chuPRSTUV* compose an operon repressed by Fur under iron sufficiency. During iron limitation (as found inside the host), high expression of the *chu* operon (for heme uptake by the transport system ChuR-ChuTUV) and the *vbaF* and *vbuA* genes (for synthesis and uptake of the siderophore viobactin) occurred (**Figures 6A, B**). Remarkably, we found that the maximum expression in iron scarcity of the TBDR genes *chuR and vbuA* depends on the small heme-binding protein ChuP. In our model, we propose that ChuP is a positive post-transcriptional regulator acting in the 5’-UTR of the *chuR and vbuA* transcripts (**Figure 6A**). Without ChuP, the expression of *chuR* and *vbuA* dropped, rendering the Δ*chuP* mutant strain its inability to use Hm and Hb *via* ChuR and its increased siderophore halos (deficiency to uptake viobactin *via* VbuA). Moreover, our virulence data in mice demonstrated that *C. violaceum* uses both heme and siderophore for iron acquisition during infection, with a preference for siderophores over the Chu heme uptake system (**Figure 6C**).

We demonstrated that the *chuPRSTUV* genes are co-transcribed from an iron-responsive and Fur-repressed promoter in *C. violaceum*, a gene cluster organization and expression pattern that fit with those found for heme uptake system in other bacteria, such as *B. multivorans*, *Yersinia* spp., and *P. aeruginosa* (Sato et al., 2017; Si et al., 2017; Schwiesow et al., 2018; Otero-Asman et al., 2019). Our nutrition assays indicated that, with the exception of *chuS*, all genes of the *chu* operon are required for heme and hemoglobin utilization in *C. violaceum*, suggesting that ChuRTUV, composed by the TBDR ChuR and the ABC transport system ChuTUV, is a heme uptake system. This mechanism of heme import across the cell envelope is found in many Gram-negative bacteria (Stojiljkovic and Hantke, 1992; Burkhard and Wilks, 2007; Balhesteros et al., 2017; Huang and Wilks, 2017). The mutant Δ*chuR* but not the mutant Δ*chuTUV* showed a small halo of heme utilization, and both mutants were unable to use hemoglobin, suggesting that *C. violaceum* maybe have another TBDR for heme uptake but relies specifically on ChuR to obtain heme from hemoglobin. Indeed, in *P. aeruginosa*, a bacterium with three heme uptake systems (Phu, Has, and Hxu), the same ABC-transport system (PhuTUV) transfers heme to the cytosol after uptake by the TBDRs PhuR and HasR (Ochsner et al., 2000; Smith and Wilks, 2015; Otero-Asman et al., 2019). ChuR appears to act as a direct heme uptake transporter given that we do not find genes encoding hemophores in *C. violaceum* and ChuR does not have an N-terminal extension typically found in hemophore-based heme uptake systems (Biville et al., 2004; Wandersman and Delepelaire, 2012; Huang and Wilks, 2017). Since the *C. violaceum* Δ*chuS* mutant showed no phenotype under heme limitation or excess, further biochemical studies are necessary to investigate whether ChuS is a heme chaperone or a non-canonical heme oxygenase involved in heme degradation, as described in other bacteria (Suits et al., 2005; Amarelle et al., 2016; Lee et al., 2017).

Recent studies have shown regulatory and functional connections between heme and siderophores (Otero-Asman et al., 2019; Batko et al., 2021; Glanville et al., 2021; Zygiel et al., 2021). The increased siderophore halos detected in Δ*chuP* and Δ*chuPRSTUV* mutant strains indicate that *chuP* is the gene of the *chu* operon that connects heme and siderophore utilization in *C. violaceum*. ChuP belongs to the HemP/HmuP protein family, whose members are found in many proteobacteria (Amarelle et al., 2019). However, only HmuP from *S. meliloti* and *B. japonicum* and HemP from *B. multivorans* have been characterized. They are small regulatory proteins required for heme utilization by acting as positive regulators of heme-acquisition TBDR genes (Amarelle et al., 2010; Escamilla-Hernandez and O’Brian, 2012; Sato et al., 2017; Amarelle et al., 2019). In *B. multivorans*, a *hmuP* mutant showed decreased siderophore halos, but the underlying mechanism remains unexplored (Sato et al., 2017). Our data indicate that ChuP links heme and siderophore utilization by acting as a positive regulator required for the expression of *chuR* and *vbuA*, genes encoding the TBDRs used by *C. violaceum* for the uptake of heme/hemoglobin (ChuR) and the siderophore viobactin (VbuA) (Batista et al., 2019). Our data favor a working model of ChuP as a heme-binding post-transcriptional regulator acting in the 5’-UTR of the *chuR and vbuA* transcripts (**Figure 6**). Supporting this model, we found that (i) ChuP of *C. violaceum* binds heme, as demonstrated for HmuP of *B. multivorans* (Sato et al., 2017); (ii) ChuP does not regulate the promoter of the *chu* operon (in front of *chuP*) and its effect on *chuR* does not occur at the transcriptional level since there is no promoter in front of *chuR* and ChuP does not bind to DNA probes covering the entire region from *chuP* to *chuR*; (iii) there is the presence, upstream of *chuR* and *vbuA*, of HPRE elements, which were described as conserved sequences probably acting on mRNA in the 5’-UTR of genes encoding heme-related TBDRs (Amarelle et al., 2019). Although HemP/HmuP proteins lack a typical DNA binding domain, they were described as direct DNA binding proteins in *B. japonicum* and *B. multivorans*, maybe by interacting with Irr and Fur (Escamilla-Hernandez and O’Brian, 2012; Sato et al., 2017). Our results suggest that ChuP in *C. violaceum* works similarly to HmuP in *E. meliloti*. However, it is necessary more work such as heme binding assays with detagged ChuP and mapping of the transcriptional start sites of *chuR* and *vbuA* to understand how ChuP binds heme and exerts its role as a post-transcriptional regulator on its target genes.

Several investigations have found that genes encoding heme uptake systems are upregulated *in vivo* (Cook et al., 2019; Rivera-Chávez and Mekalanos, 2019) and required for colonization and virulence of many bacterial pathogens (Skaar et al., 2004; Si et al., 2017; Abdelhamed et al., 2018; Cook et al., 2019; Rivera-Chávez and Mekalanos, 2019; Chatterjee et al., 2020). In many cases, bacteria explore multiple host iron sources, employing both heme and siderophore-based iron acquisition systems (Contreras et al., 2014; Huang and Wilks, 2017). Our prior work revealed that *C. violaceum* requires catecholate-type siderophores for virulence in mice (Batista et al., 2019). Our current findings based on the characterization of mutants without either siderophores, the *chu* operon, or both indicate that *C. violaceum* uses siderophores and heme but prioritizes siderophores over heme as an iron source during infection, at least in our mice model of acute systemic infection (**Figure 6C**). In agreement with our data, a study that characterized mutants of multiple iron uptake systems showed a clear predominance of siderophores over heme transport systems in *P. aeruginosa* infecting lung (Minandri et al., 2016). However, the preference for a particular iron source changes according to its availability or the infection context. For instance, *S. aureus* prefers heme but uses siderophores when heme is scarce (Skaar et al., 2004); *P. aeruginosa* prioritizes siderophore systems in acute infections but switches to heme in long-term chronic infections (Marvig et al., 2014; Nguyen et al., 2014); and *Vibrio cholerae* relies on heme released by cholera toxin-dependent damage in the intestine (Rivera-Chávez and Mekalanos, 2019). Currently, we are developing a mouse model of abscess for *C. violaceum* infection. It will be interesting to investigate in this model whether *C. violaceum* alters its preference for siderophores and heme in log-term infections.

## Data Availability Statement

The original contributions presented in the study are included in the article/**Supplementary Material**. Further inquiries can be directed to the corresponding author.

## Ethics Statement

The animal study was reviewed and approved by Local Ethics Animal Committee (CEUA), Faculdade de Medicina de Ribeirão Preto, Universidade de São Paulo.

## Author Contributions

JFdSN and VL conceived and designed the study. VL and BB performed the experiments. VL and JFdSN performed data analysis and interpretation. VL and JFdSN wrote the paper. All authors contributed to the article and approved the submitted version.

## Funding

This research was supported by grants from the São Paulo Research Foundation (FAPESP; grants 2018/01388-6 and 2020/00259-8) and Fundação de Apoio ao Ensino, Pesquisa e Assistência do Hospital das Clínicas da FMRP-USP (FAEPA). During this work VL (2018/17716-2) and BB (2018/19058-2) were supported by FAPESP fellowships.

## Conflict of Interest

The authors declare that the research was conducted in the absence of any commercial or financial relationships that could be construed as a potential conflict of interest.

## Publisher’s Note

All claims expressed in this article are solely those of the authors and do not necessarily represent those of their affiliated organizations, or those of the publisher, the editors and the reviewers. Any product that may be evaluated in this article, or claim that may be made by its manufacturer, is not guaranteed or endorsed by the publisher.
