# Supplementary material for: The Regulatory Protein ChuP Connects Heme and Siderophore-Mediated Iron Acquisition Systems Required for Chromobacterium violaceum Virulence
Source: Front Cell Infect Microbiol. 2022 May 11;12:873536. doi: 10.3389/fcimb.2022.873536 (PMC9131926; doi:10.3389/fcimb.2022.873536)
Supplement: Supplementary file 1 [file DataSheet_1.pdf]

Supplementary Material

The regulatory protein ChuP connects heme and siderophore-mediated iron acquisition systems required for *Chromobacterium violaceum* virulence

Vinicius M. de Lima[1], Bianca B. Batista[1] and José F. da Silva Neto[1*]

[1]Departamento de Biologia Celular e Molecular e Bioagentes Patogênicos, Faculdade de Medicina de Ribeirão Preto, Universidade de São Paulo, Ribeirão Preto, SP, Brazil

***Correspondence:**

José F. da Silva Neto

jfsneto@usp.br

Table S1. Primers used in this work

Primer name	Sequence (5'→3')[a]	Description
**Construction of mutant strains**		
chuP_del1[b]	taccggaagcttcccagctgtagtcgatgatg	*Hind*III/*Bam*HI upstream flanking fragment with 656 bp
chuP_del2[b]	taccggggatccgggcggtgagtgatatgtgc	
chuP_del3	taccggggatccatcaagtaacacccgcaagcc	*Bam*HI/*Eco*RI downstream flanking fragment with 599 bp
chuP_del4	taccgggaattcttgacgctgccgcctatccc	
chuR_del1	taccggaagcttcccttgcgtgtttcctgcgc	*Hind*III/*Bam*HI upstream flanking fragment with 628 bp
chuR_del2	taccggggatccgacctggatggtctccagcg	
chuR_del3[3]	taccggggatcccagaccgccggtttcgagac	*Bam*HI/*Eco*RI downstream flanking fragment with 672 bp
chuR_del4	taccgggaattcttgtgcacggcttctccgtg	
chuS_del1	taccggaagcttagtaccagaacatcggccgg	*Hind*III/*Bam*HI upstream flanking fragment with 642 bp
chuS_del2[4]	taccggggatcccagctcgcattcgctgacgc	
chuS_del3	taccggggatccgagcgggcggaatgggtgaa	*Bam*HI/*Eco*RI downstream flanking fragment with 632 bp
chuS_del4[6]	taccgggaattcccagcggcttgaaaccgttg	
chuTUV_del1	taccggaagcttagcctgaacgacgtgcacgc	*Hind*III/*Bam*HI upstream flanking fragment with 634 bp
chuTUV_del2	taccggggatcccagctcggcttccttgctgc	
chuTUV_del3[b]	taccggggatccaagaccgtcgcccaggtgct	*Bam*HI/*Eco*RI downstream flanking fragment with 678 bp
chuTUV_del4[b]	taccgggaattcgcgaagaatacccgctggtg	
M13_FW	gtaaaacgacggccagt	Sequencing of cloned fragments
M13_RV	agcggataacaatttcac	
**Construction of complemented strains**		
chuP_CompFW[c]	taccggggtacctccccttgcgtgtttcctgc	*Kpn*I/*Eco*RI 631 bp fragment with *chuP* and its promoter
chuP_CompRV	taccgggaattctgacctggatggtctccagc	region
chuR_CompFW	taccggggtaccagtaacacccgcaagccagc	*Kpn*I/*Eco*RI 2423 bp fragment with *chuR*
chuR_CompRV	taccgggaattcaccagctcgcattcgctgac	
chuS_CompFW	taccggggtaccccaattctgatccacccggc	*Kpn*I/*Eco*RI 1195 bp fragment with *chuS*
chuS_CompRV	taccgggaattccgactacgatctcggcgatg	
chuTUV_CompFW[5]	taccggggtaccagcgggcggaatgggtgaag	*Kpn*I/*Eco*RI 2633 bp fragment with *chuTUV*
chuTUV_CompRV[c]	taccgggaattcctacgcgctgaagtaaggcg	
**Heterologous expression**		

chuP_ExpFW	taccggcatatgagcacatatcactcaccgc	*Ndel*/*Bam*HI 174 bp fragment with *chuP* open reading frame
chuP_ExpRV	taccggggatccttacttgatcagtatcagtttgcc	
T7_promoter	taatacgactcactataggg	Sequencing of cloned gene
T7_terminator	gctagttattgctcagcgg	
**β-galactosidase assay and EMSA**		
chuP_promot_FW[d]	taccgggaattcgcttgccgaaggtcagcg	*Eco*RI/*Hind*III 564 bp fragment with *chuP* promoter region for pRK*lacZ*290 cloning
chuP_promot_Rv	taccggaagcttgctgcaggcggtagatctc	
chuR_promot_FW[1]	taccgggaattctgagcacatatcactcaccg	*Eco*RI/*Hind*III 439 bp fragment upstream of *chuR* for pRKlacZ290 cloning
chuR_promot_RV[2,d]	taccggaagcttttcgccgcgtcggcaatatc	
CV_2599_promotFW	cctagcgaattcgcggccaaagagtcaggaa	*Eco*RI/*Bam*HI 288 bp fragment with CV_2599 promoter region
CV_2599_del2	ggcctaggatcctacccgttgtacggcagcg	
l*acZ*290up	tgacggctatcaccatca	Confirmation of cloned sequences into pRK*lacZ*290
**RT-qPCR**		
CV_3896NB_FW	gaccttccccagcaagaccttc	124 bp fragment of *chuR* coding region
CV_3896NB_RV	cttgtaatggtcccagcgcagc	
vbaF_RT-qPCR_FW	cgctgcagtacggactgga	110 bp fragment of *vbaF* coding region
vbaF_RT-qPCR_RV	tatggtgtcggcgccatac	
vbuA_RT-qPCR_FW	cctgaacatgcgtttggacg	122 bp fragment of *vbuA* coding region
vbuA_RT-qPCR_FW	gcaggttttggtcacgatcg	
CV_4206NB_FW	gaaaaaccgctcttcatgctg	122 bp fragment of *rpoH* coding region
CV_4206NB_FW	gatcgtggttgccgctgaaa	

[a] Digestion sites recognized by the restriction enzymes are underlined.

[b]These primers were also used to delete the entire *chu* operon.

[c]These primers were also used to complement the entire *chu* operon.

[d] These primers were also used to generate the p*chuPR* EMSA probe.

[1-6]Primers employed in RT-PCR.

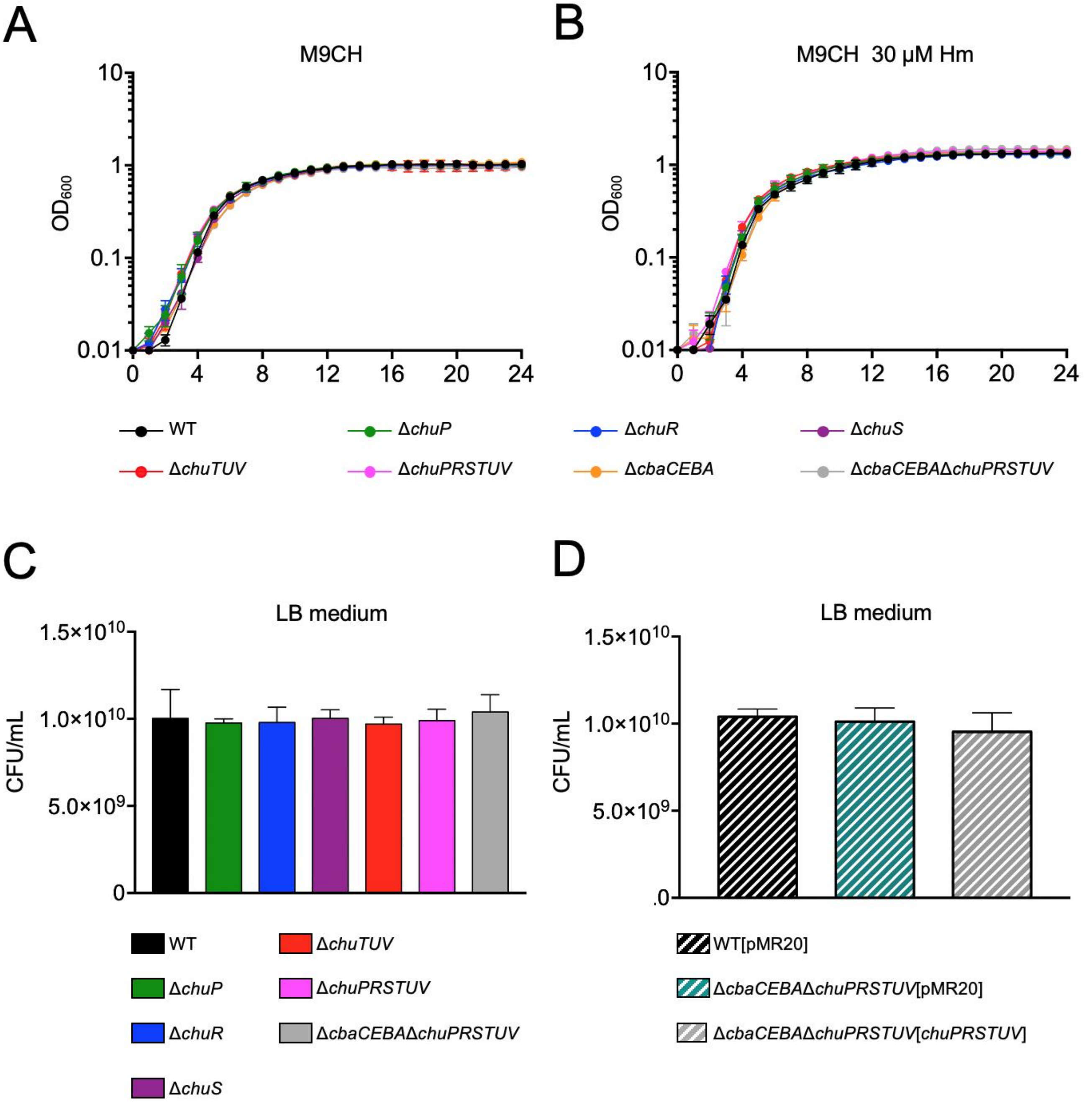

Supplementary Figure 1. Mutants of the *chu* operon have regular fitness under standard growth conditions. **(A and B)** Growth curves. The WT and the indicated mutant strains were grown in M9CH without **(A)** or with Hm supplementation **(B)** from an OD_{600} of 0.01. The OD_{600} was measured every 15 minutes for 24 h. Data points are shown in 1-h intervals as the mean and standard deviation of three biological replicates. **(C and D).** Cell viability in LB medium by CFU counting. The indicated mutant **(C)** and complemented **(D)** strains were grown for 20 h in LB, serial diluted, and plated for CFU quantification. Data are from three biological replicates. Mutant and complemented strains were compared to WT and WT[pMR20], respectively. When not indicated, n.s. (not significant). One-way ANOVA followed by Tukey's multiple-comparison test.

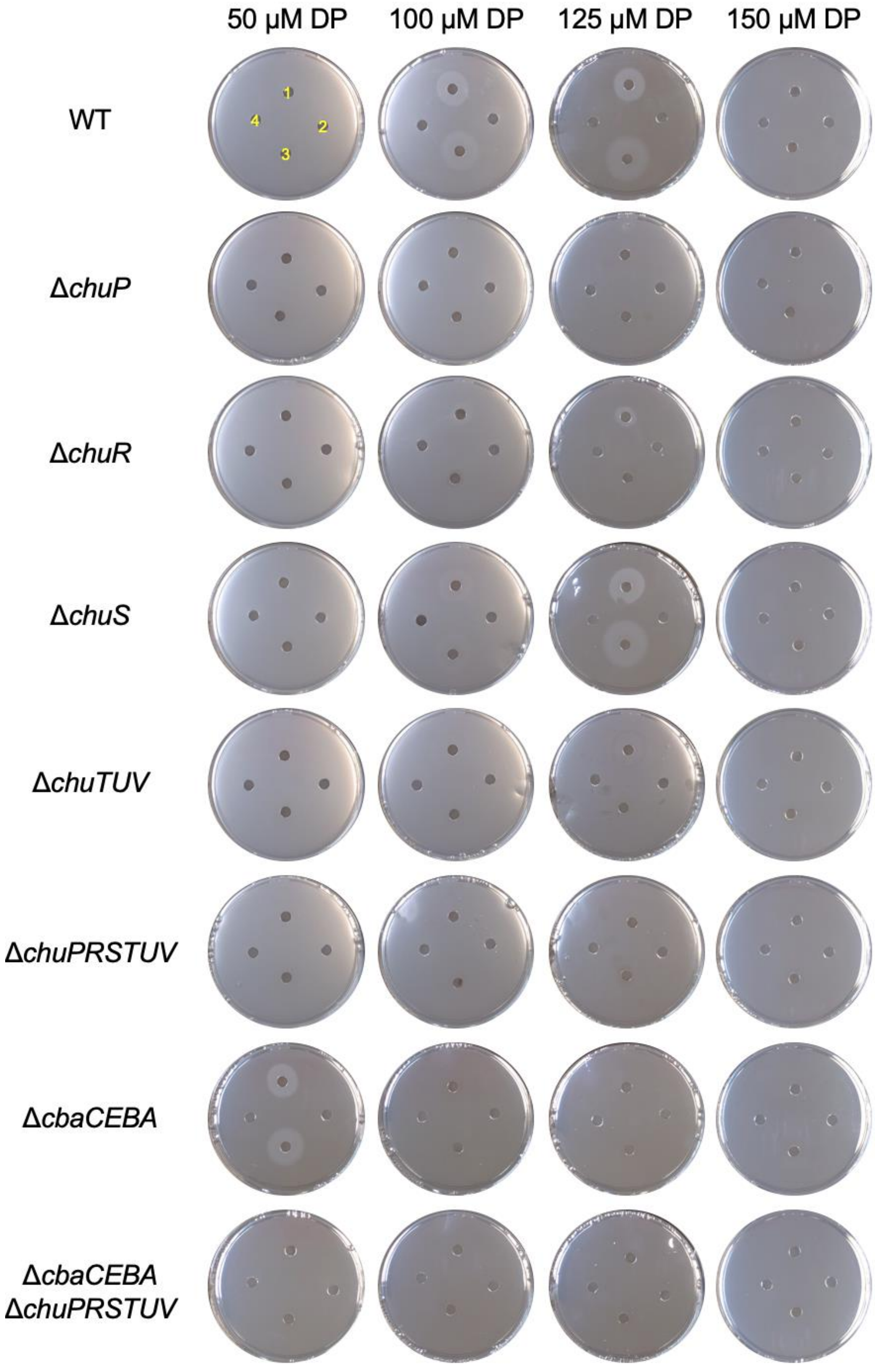

Supplementary Figure 2. Nutrition assays for Hm and Hb under different conditions of DP-imposed iron deficiency. The indicated strains were embedded into M9CH medium supplemented with the indicated DP concentrations. Aliquots of Hm and Hb were provided as iron sources. Growth halos around the discs indicate compound utilization. Representative images are shown. 1 - 100 μM Hm; 2 - 20 mM NaOH; 3 - 150 μM Hb; 4 - 100 mM NaCl.

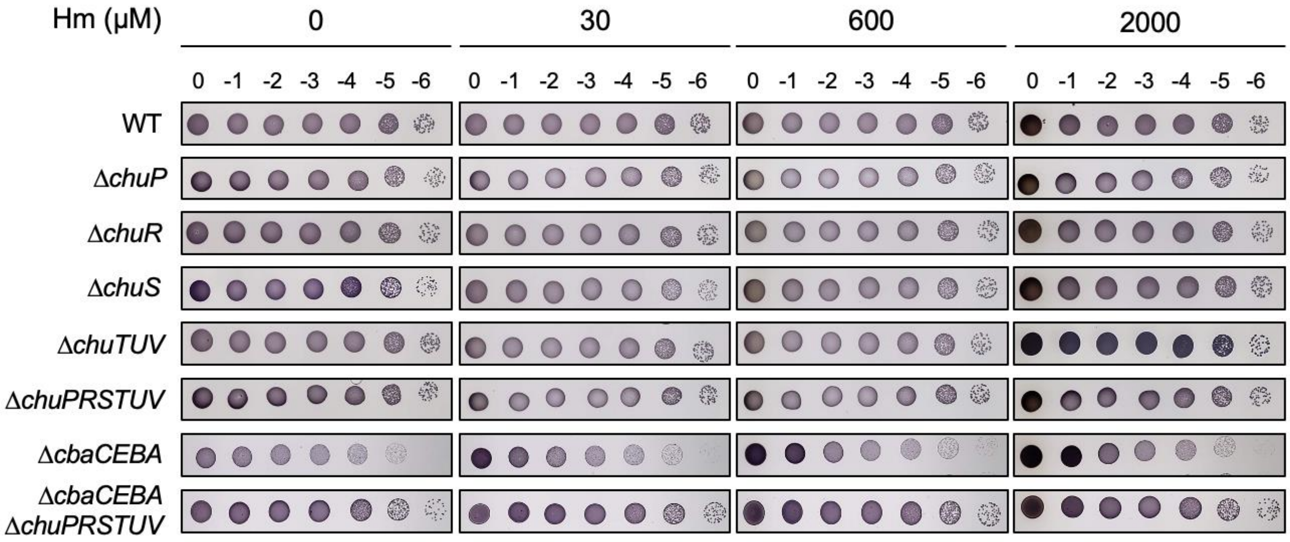

Supplementary Figure 3. Mutants of the *chu* operon have regular fitness under heme excess. *C. violaceum* WT and the indicated mutant strains were grown from an OD_{600} of 0.01 in either M9CH or M9CH supplemented with the indicated Hm concentrations. After 24 h cultivation, the cultures were serial diluted and platted on M9CH to evaluate cell viability. Data representative of three biological replicates.

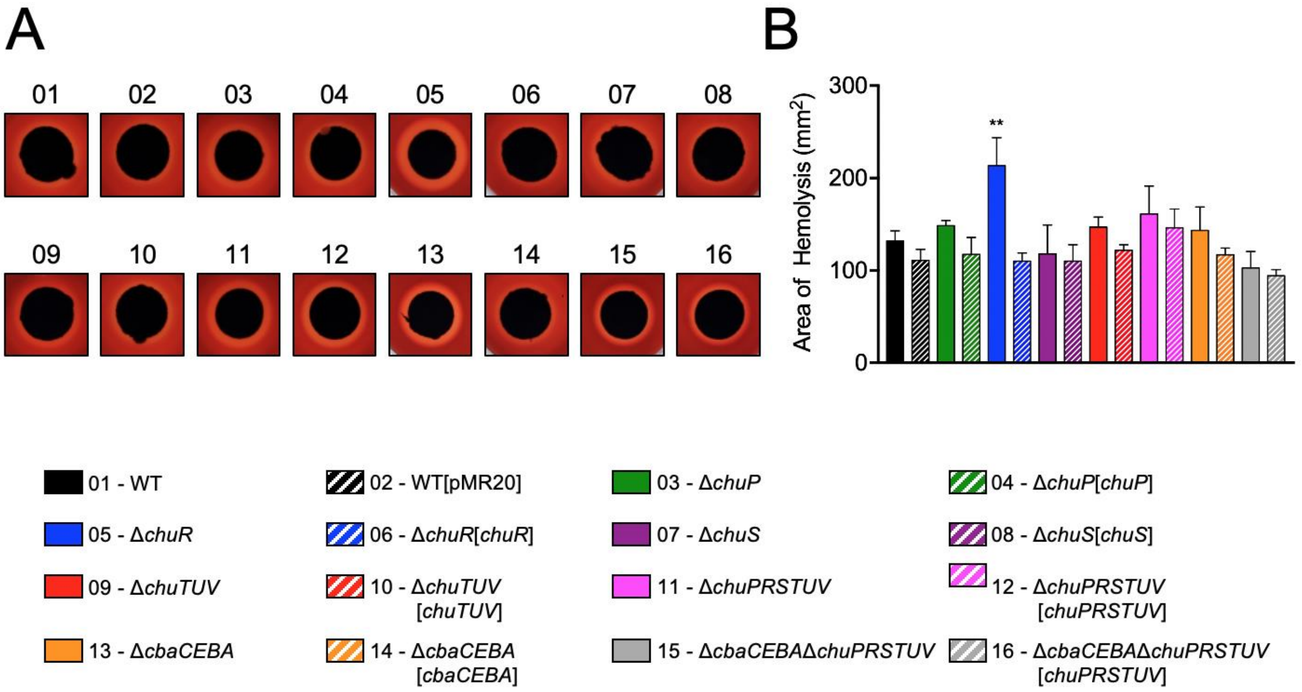

Supplementary Figure 4. Deletion of *chuR* increases hemolytic activity. **(A)** Hemolysis in blood agar. The indicated strains were grown in M9CH and spotted on 5% (v/v) sheep-blood Mueller-Hinton plates. The lighter halos around bacteria growth indicate hemolysis. Representative images of one assay are shown. **(B)** Quantification of hemolytic activity. The area of the lighter halos was measured using Image J software, subtracting the area of bacterial growth to eliminate differences in sizes. Data are from three biological replicates. Mutant and complemented strains were compared to WT and WT[pMR20], respectively. **, $p < 0.01$; when not shown, n.s. (not significant). One-way ANOVA followed by Tukey's multiple-comparison test.
